# Noninvasive characterization of peripheral sympathetic activation across sensory stimuli using a peripheral arterial stiffness index

**DOI:** 10.3389/fphys.2023.1294239

**Published:** 2024-01-08

**Authors:** Ziqiang Xu, Reiji Anai, Harutoyo Hirano, Zu Soh, Toshio Tsuji

**Affiliations:** ^1^ Graduate School of Advanced Science and Engineering, Hiroshima University, Hiroshima, Japan; ^2^ Department of Medical Equipment Engineering, Clinical Collaboration Unit, School of Medical Sciences, Fujita Health University, Toyoake, Aichi, Japan

**Keywords:** noninvasive characterization, peripheral arterial stiffness index, peripheral sympathetic activation, sensory stimuli, transient response analysis

## Abstract

**Introduction:** The peripheral arterial stiffness index has been proposed and validated as a noninvasive measure quantifying stimulus intensity based on amplitude changes induced by sympathetic innervation of vascular tone. However, its temporal response characteristics remain unclear, thus hindering continuous and accurate monitoring of the dynamic process of sympathetic activation. This paper presents a study aimed at modeling the transient response of the index across sensory stimuli to characterize the corresponding peripheral sympathetic activation.

**Methods:** The index was measured using a continuous arterial pressure monitor and a pulse oximeter during experiments with local pain and local cooling stimuli designed to elicit different patterns of sympathetic activation. The corresponding response of the index was modeled to clarify its transient response characteristics across stimuli.

**Results:** The constructed transfer function accurately depicted the transient response of the index to local pain and local cooling stimuli (Fit percentage: 78.4% ± 11.00% and 79.92% ± 8.79%). Differences in dead time (1.17 ± 0.67 and 0.99 ± 0.56 s, *p* = 0.082), peak time (2.89 ± 0.81 and 2.64 ± 0.68 s, *p* = 0.006), and rise time (1.81 ± 0.50 and 1.65 ± 0.48 s, *p* = 0.020) revealed different response patterns of the index across stimuli. The index also accurately characterized similar vasomotor velocities at different normalized peak amplitudes (0.19 ± 0.16 and 0.16 ± 0.19 a.u., *p* = 0.007).

**Discussion:** Our findings flesh out the characterization of peripheral arterial stiffness index responses to different sensory stimuli and demonstrate its validity in characterizing peripheral sympathetic activation. This study valorizes a noninvasive method to characterize peripheral sympathetic activation, with the potential to use this index to continuously and accurately track sympathetic activators.

## 1 Introduction

The sympathetic nervous system (SNS) responds to environmental cues and innervates peripheral tissues in a highly selective manner, maintaining homeostasis precisely and cost-effectively Kregel et al. (1992); Boron and Boulpaep (2017); Greaney and Kenney (2017). For example, mild arousal induces a reduction in cutaneous blood flow to maintain cerebral oxygenation and sensory focus, and intense emotional stress elicits an increase in skeletal muscle blood flow to prepare for a defense reaction, the latter commonly known as the “fight-or-flight” response Kiyatkin (2021). Whereas the valence and intensity of the stimulus determine differential sympathetic outflow to the target tissue, tissue-specific behaviors like vasomotion, sudomotion, cardiac activation, and thermogenesis also reflect the dynamics of their respective sympathetic innervation Morrison (2001); Mano et al. (2006); Hu et al. (2020); Kandel et al. (2021). Accordingly, relevant behavior-dependent markers such as vascular resistance, blood flow, arterial blood pressure (ABP), heart rate (HR), sweat, and skin resistance are employed to identify and evaluate sympathetic activity as well as higher cortical activity elicited by various stimuli. Furthermore, the responsivity of these behavior-dependent markers enables the detection and examination of functional abnormalities in the corresponding target tissues Mano et al. (2006); Silverthorn and Michael (2013); Raj et al. (2022). Therefore, cues of stimuli and underlying pathologies can be evaluated using these markers, thus requiring that their responses accurately characterize the corresponding sympathetic activators.

Among these markers, hemodynamic indices, such as HR and ABP, serve as representative measures to be analyzed to evaluate sympathetic activation in clinical and daily settings owing to their noninvasive measurability Shaffer and Ginsberg (2017); Rosei et al. (2020); Schutte et al. (2022); Raj et al. (2022). However, both HR and ABP primarily reflect systemic changes in macrocirculation, rendering them less sensitive to mild stimuli and susceptible to the control of multiple simultaneous stimuli Xu et al. (2022b). Consequently, these factors pose challenges for accurately characterizing sympathetic activation, particularly in peripheral tissues involving microcirculation. On the other hand, the impetus to accurately monitor peripheral sympathetic activity in the clinic has given rise to a neurophysiological method called microneurography Greaney and Kenney (2017); Mano et al. (2006); Vallbo and Hagbarth (1968); Delius et al. (1972); Iwase et al. (2017). This invasive technique involves direct recording of muscle and cutaneous fascicles of peripheral sympathetic nerves by inserting a microelectrode into the skin of a conscious participant. Consequently, it enables separate measurement of sympathetic burst activity in the skin (skin sympathetic nerve activity, SSNA) and skeletal muscle (muscle sympathetic nerve activity, MSNA). Since the 1960 s, this technique has significantly expanded our understanding of the pattern of sympathetic postganglionic outflow to peripheral tissues. However, the simultaneous measurement of SSNA and MSNA with this technique is challenging and requires extensive training. Therefore, the invasive and labor-intensive nature of this technique imposes a burden on both participants and experimenters, promoting us to adopt a convenient and accurate method to characterize peripheral sympathetic activation.

Given that vasomotion is involved in most of the hemodynamic consequences of sympathetic activation, our research group proposed a noninvasive index of peripheral arterial stiffness to estimate the sympathetic elevation of vascular tone Sakane et al. (2003). This index is derived from an approximate model depicting the nonlinear relationship between relative changes in arterial blood volume and distending pressure, which is obtained by simultaneous measurement of a finger photoplethysmogram (PPG) and continuous arterial pressure. A panoply of demonstrations and related applications based on this index has been launched in previous studies Hirano et al. (2013); Elbegzaya et al. (2017); Muneyasu et al. (2021); Matsubara et al. (2018); Tsuji et al. (2021); Kamiya et al. (2021). For example, this index can effectively monitor changes in vascular tone during endoscopic thoracic sympathectomy, surpassing the direct monitoring of changes in blood flow using PPG and laser Doppler flowmetry Hirano et al. (2013); Elbegzaya et al. (2017); Muneyasu et al. (2021). In addition, this index allows for quantitative assessment of subjective pain sensation, and its pain-elicited changes have been demonstrated to correlate positively with changes in brain activity associated with pain perception Matsubara et al. (2018); Tsuji et al. (2021). However, given the dynamic innervating role of the SNS in response to stimuli, it is necessary to model and clarify the response characteristics of the index for further accurate assessment. Methodologically, the index enables differential responses to diverse levels of sympathetic activation in the dimensions of responsivity and response frequency Tsuji et al. (2021); Muneyasu et al. (2021). Consequently, its transient responses may incorporate rich information on environmental cues, offering the potential for accurately characterizing peripheral sympathetic activation.

This paper aims to model the response of the peripheral arterial stiffness index to different stimuli and verify its ability to characterize peripheral sympathetic activation. Here, experiments involving local pain and local cooling stimuli were conducted to evoke distinct sensations and thus elicit different responses from this index. Furthermore, considering the presence of vasomotor and sudomotor activity at peripheral sites, we concurrently modeled the transient responses of this index and palm sweat rate and analyzed their response characteristics across stimuli. The timescale and waveform characteristics of the index in response to the two stimuli were analyzed to confirm its characterization ability.

## 2 Materials and methods

**FIGURE 1:**
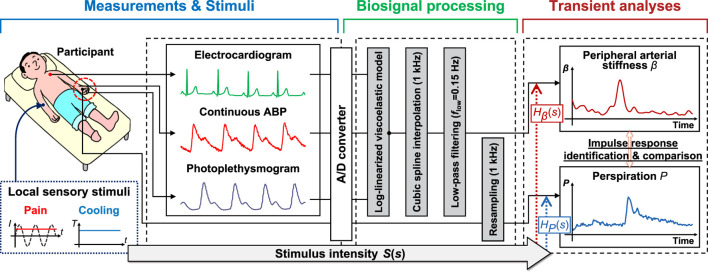
Overview of the method for analyzing the response characteristics of the peripheral arterial stiffness index to sensory stimuli. The method comprises three parts: Measurements and stimuli, biosignal processing, and transient analyses. *I*: stimulus current intensity; *T*: stimulus temperature; ABP: arterial blood pressure; *S*(*s*): the Laplace transform of stimulus intensity; *H*_*β*_(*s*) and *H*_*P*_(*s*): transfer functions of peripheral arterial stiffness index *β* and palm sweat rate *P*, respectively.

Figure 1 presents an overview of the study, which analyzes the response of the peripheral arterial stiffness index when exposed to local pain and local cooling stimuli; this comprises three parts: measurements and stimuli, biosignal processing, and transient analyses. Utilizing the system identification method to estimate the impulse response, the transient response characteristics of the palm sweat rate and the stiffness index in response to local sensory stimuli can be derived respectively. By comparing these characteristics, it is possible to investigate the patterns of peripheral sympathetic activation as assessed by the stiffness index under different stimuli.

### 2.1 Study design and participants

In this study, local pain and local cooling stimuli were designed to evoke different somatosensations and elicit different responses in peripheral target organs innervated by the SNS. Since sweating is a clear and distinctive marker of sudomotor activity in response to thermal changes and arousal stimuli Kuwahara et al. (2015), its response can serve as a reference for peripheral sympathetic activation. In the local pain stimulus experiment, the desired pain sensation was achieved by setting the current intensity to a level where participants verbally reported a pain level of “50” on the visual analog scale (VAS). This approach was intended to generate sufficient pain while minimizing interindividual differences in pain perception Matsubara et al. (2018); Tsuji et al. (2021); Melzack and Raja (2005); Xu et al. (2022a). The local cooling stimulus experiment aimed to evoke a sensation of coldness, with a fixed temperature of 10°C for local cold exposure. Previous reports indicate that at this temperature, the skin temperature at the stimulated area drops from 35°C to approximately 21°C, then returns to about 25°C within 15 s post-stimulus Torato et al. (2016). These temperatures are above the human pain threshold of 18°C, ensuring that most participants would not experience pain Iwase et al. (2017); Rossi and Neubert (2009). Moreover, both skin cooling (<25°C) and pain sensations are known to be transmitted through Aδ nerve fibers Kandel et al. (2021), leaving the potential for variations in the pattern of peripheral sympathetic activation. The primary objective of this study is to ascertain whether the peripheral arterial stiffness index can accurately characterize different patterns of peripheral sympathetic activation elicited by these different sensory stimuli.

We studied the response characteristics of the peripheral arterial stiffness index and palm sweat rate in 20 healthy young adults (Japanese males, age: 22.05 ± 0.92 years [mean ± S.D.]). All experiments were conducted in accordance with the principles of the Declaration of Helsinki. Informed consent was obtained from all study participants before conducting the experiments, and the study was approved by the Hiroshima University Ethics Committee (Registration number: E-17-2).

### 2.2 Experimental configurations

All participants were studied in the supine position within a thermoneutral environment. Participants were surrounded by shade cloth and equipped with noise-canceling headphones to avoid postural changes and minimize external distractions that might additionally activate the SNS.

#### 2.2.1 Protocol and procedures

**FIGURE 2:**
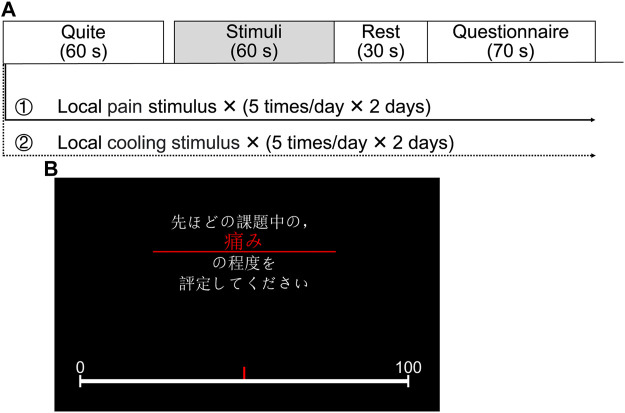
Experimental configurations. **(A)** Experimental protocol. **(B)** A questionnaire displayed on the screen facing the participant (Japanese text in the image: “Please evaluate the degree of pain during the previous stimulus.”).

The experimental protocol, as shown in Figure 2A, commenced with a 60-s rest to relax the participants before each experiment. This was followed by the execution of the experimental task, starting with a 60-s sensory stimulus to activate the SNS and then a 30-s rest period. The task ended with a 70-s questionnaire, comprising seven items (pain, heat, cold, pleasure, unpleasantness, arousal, and calm), presented in random order, totaling a duration of 230 s for each experiment. To prevent sensory inactivation due to multiple stimuli in a short period, the same stimulus was repeated five times in 1 day for each participant, and the two experiments were spread over 4 days. In this study, the questionnaire on subjective sensations was carried out using the VAS based on a 100-mm graduated line mark, with the leftmost 0 representing “no pain” and the rightmost 100 representing “the worst pain imaginable” as shown in Figure 2B. Here, a fixed stimulus intensity was set for each task to streamline the system identification analyses. The interval between two consecutive experiments was sufficiently long to ensure that the participant’s skin temperature returned to its initial value and that they reported no lingering abnormalities.

#### 2.2.2 Measurements and stimuli

To analyze the transient responses of the peripheral arterial stiffness index and palm sweat rate, several measurements were taken simultaneously during the experiment, including ECG, noninvasive continuous ABP, fingertip PPG, and palm sweat rate. A three-lead ECG and left radial artery pressure signals were measured using a biological information monitor (BP-608EVII CS, FUKUDA COLIN Co., Ltd., Tokyo, Japan). The ECG was measured to provide cardiac cycle timing for estimating the index beat by beat. The accuracy of ABP measurement with this device has been validated against a mercury sphygmomanometer, the gold-standard instrument for office BP measurements Chahine et al. (2015). The PPG signal was measured using a pulse oximeter (OLV-4202, NIHON KOHDEN Corp., Tokyo, Japan). The palm sweat rate was measured using a digital perspiration meter (SMN-1000, SKINOS Co., Ltd., Nakano, Japan), which has been proven effective in detecting changes in palm sweat rate elicited by mental stress Zheng et al. (2015). All the biosignals were collected via BNC connectors, fed into an analog/digital (A/D) converter (DC-300H, NIHON KOHDEN Corp., Tokyo, Japan), and visualized and recorded using LabChart 8 software (ADInstruments Pty. Ltd., Dunedin, New Zealand). The sampling frequency for all biosignals was set at 2 kHz.

The local pain stimulus was generated using an electrocutaneous stimulus system, which comprises an isolator (SS-203J, NIHON KOHDEN Corp., Tokyo, Japan), an analog function generation (WF 1973, NF Corporation, Kanagawa, Japan), an electric stimulator (SEN-3401, NIHON KOHDEN Corp., Tokyo, Japan), and a surface stimulation electrode (NM-990W, NIHON KOHDEN Corp., Tokyo, Japan). In the local pain stimulus experiment, a 250 Hz sinusoidal electrocutaneous stimulus of predetermined intensity was applied to the participant’s right forearm to evoke pain and activate the SNS. For the local cooling stimulus, the same skin area was exposed to a skin cooling device (PT10, Creative Medical Engineering Co, Ishikawa, Japan), featuring a 3.5 cm diameter circular cooling zone. Skin temperature was confirmed before and after each stimulus using an imaging infrared thermometer (TG167, Teledyne FLIR LLC, Oregon, United States).

### 2.3 Biosignal processing method

#### 2.3.1 Peripheral arterial stiffness

As previously stated, our research group focused on the changes in the mechanical impedance of the arterial wall during peripheral vasomotion and proposed an assessment index based on a log-linearized peripheral arterial viscoelastic model. Considering the viscoelastic nature of arteries, the corresponding changes in arterial volume and mechanical properties in response to a radial force exerted on the arterial wall can be modeled as:

$$\begin{array}{cc} F\left(t\right) & =F_{\mu }\left(t\right)+F_{\eta }\left(t\right)+F_{\beta }\left(t\right)\\ & \approx \tilde{\mu }\ddot{r}\left(t\right)+\tilde{\eta }\dot{r}\left(t\right)+\exp\left\{\tilde{\beta }dr\left(t\right)+F_{{\tilde{\beta }}_{0}}+F_{{\tilde{\beta }}_{nl}}\left(r\left(t\right)\right)\right\}, \end{array}$$ (1)

where *F*(*t*), *F*_*μ*_(*t*), *F*_*η*_(*t*), and *F*_*β*_(*t*) denote the radial force applied to the arterial wall, the inertial force and internal frictional force arising from the blood flow, and the force generated by the arterial stiffness to resist deformation arising from the applied force, respectively. $\tilde{\mu }$ , $\tilde{\eta }$ , and $\tilde{\beta }$ represent the inertia, viscosity, and stiffness of the arterial wall, respectively. *r*(*t*), $\dot{r}\left(t\right)$ , $\ddot{r}\left(t\right)$ , and *dr*(*t*) denote the arterial diameter and the instantaneous change velocity, acceleration, and value of the arterial diameter, respectively. *t* represents time and the dot operation on *r*(*t*) represents the time derivative. exp $\left\{\cdot \right\}$ represents a logarithmic relationship between the force generated by the arterial stiffness and the resulting deformations modulated by multiple factors, including circulatory filling pressure, peripheral reflection, and sympathetic innervation. Correspondingly, $F_{{\tilde{\beta }}_{0}}$ represents the force originating from circulatory filling pressure and $F_{{\tilde{\beta }}_{nl}}\left(r\left(t\right)\right)$ represents the force originating from peripheral reflection.

For the sake of simplicity, ABP is used to substitute the radial force *F*(*t*) on the basis of one unit area of the arterial wall. In addition, since the deformation *dr*(*t*) of the arterial wall is difficult to measure directly, PPG is applied here because it can approximately linearly reflect the change of arterial volume by measuring the hemoglobin concentration in the artery. Consequently, Eq. 1 is redefined as:

$$P_{b}\left(t\right)=\mu {\ddot{P}}_{l}\left(t\right)+\eta {\dot{P}}_{l}\left(t\right)+\exp\left\{\beta P_{l}\left(t\right)+P_{b\beta_{0}}+P_{b\beta_{nl}}\left(P_{l}\left(t\right)\right)\right\},$$ (2)

where *P*_*b*_(*t*), *P*_*l*_(*t*), $\dot{P_{l}}\left(t\right)$ , and $\ddot{P_{l}}\left(t\right)$ represent the arterial blood pressure and the instantaneous change value, velocity, and acceleration of PPG at time *t*, respectively. $P_{b\beta_{0}}$ represents zero transmural pressure and $P_{b\beta_{nl}}\left(P_{l}\left(t\right)\right)$ represents the pressure originating from the distal reflected pressure. Here, the viscoelasticity parameters *μ*, *η*, and *β* for each heartbeat cycle can be estimated using the linear least squares method Matsubara et al. (2018).

#### 2.3.2 Pre-processing method

Since the peripheral arterial stiffness index *β* in Eq. 2 is computed beat-by-beat and generated only at the R-peak times of the ECG, cubic spline interpolation is performed within two adjacent R-peak times to construct a continuous curve with a frequency of 1 kHz. Then, considering that the respiratory modulation of the cardiovascular system falls within the frequency range of 0.16–0.33 Hz Xu et al. (2022b); Russo et al. (2017), a low-pass filter with a cut-off frequency of 0.15 Hz is subjected to the interpolated results of *β*. Thus, the low-frequency component is assumed to be the acute value of arterial stiffness, reflecting changes elicited solely by external sensory stimuli and/or mental stress. Additionally, the palm sweat rate data is resampled from 2 kHz to 1 kHz to match the frequency of *β* and reduce the computational load. All data are normalized with a mean of 0 and a variance of 1 to pool and scale numeric features and minimize inter- and intra-individual differences.

### 2.4 Data analyses

#### 2.4.1 Transient analysis

Here, the input-output relationship between the stimulus intensity and the two indices can be described using the transfer function model:

$$\beta \left(s\right)=H_{\beta }\left(s\right)S\left(s\right),$$ (3)

$$P\left(s\right)=H_{p}\left(s\right)S\left(s\right),$$ (4)

where *S*(*s*), *β*(*s*), *P*(*s*), *H*_*β*_(*s*), and *H*_*p*_(*s*) denote the Laplace transform of stimulus intensity, peripheral arterial stiffness index, and palm sweat rate, and transfer functions of these two indices to a stimulus, respectively. Here, according to the positive correlation between stimulus intensity, subjective sensation, and physiological measures Matsubara et al. (2018); Tsuji et al. (2021); Xu et al. (2022a), the input used for system identification was the pre-determined current intensity from the local pain stimulus experiment and the cold sensation reported verbally by the participant in the local cooling stimulus experiment. The two normalized indices *β*_n_ and *P*_n_ over 50–80 s were used as the output to identify transfer functions.

In this study, the function “tfest” from the system identification toolbox in MATLAB was employed to identify transfer functions comprising a specified number of poles and zeros, along with an unknown time delay, from the collected input-output data Garnier et al. (2003); Ljung (2019). Moreover, the mean squared error (MSE), fit percentage (FP), Akaike information criterion (AIC), and Bayesian information criterion (BIC) were employed to evaluate the quality of the identification results, which are defined as:

$$MSE=\frac{1}{N}\overset{N}{\underset{i=1}{\sum }}{\left(\;{\hat{y}}_{i}-y_{i}\right)}^{2},$$ (5)

$$FP=100\%\left(1-\frac{\sqrt{MSE}}{y_{\max}-y_{\min}}\right),$$ (6)

$$AIC=N\ln\frac{1}{N}\overset{N}{\underset{i=1}{\sum }}{\left(\;{\hat{y}}_{i}-y_{i}\right)}^{2}+2n_{p}+N\left(\ln2\pi +1\right),$$ (7)

$$BIC=N\ln\frac{1}{N}\overset{N}{\underset{i=1}{\sum }}{\left(\;{\hat{y}}_{i}-y_{i}\right)}^{2}+N\left(\ln2\pi +1\right)+n_{p}\ln N,$$ (8)

where ${\hat{y}}_{i}$ , *y*_*i*_, *N*, *y*_max_, *y*_min_, and *n*_*p*_ denote the estimated value, measured value, number of values in the estimation data set, maximum and minimum value of the measured value, and number of free parameters, respectively.

**FIGURE 3:**
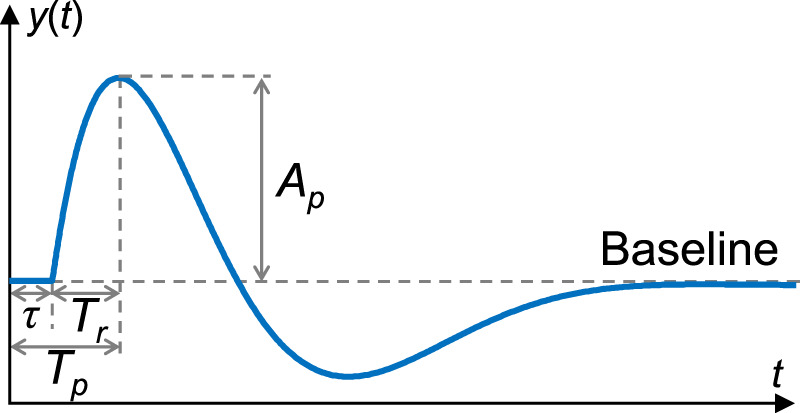
An example of impulse response and its characteristics analyzed in this study. The baseline indicates a steady state, i.e., the rest state. *y*(*t*): Impulse response; *t*: Time; *τ*: Dead time; *T*_*p*_: Peak time; *T*_*r*_: Rise time; *A*_*p*_: Baseline-to-peak amplitude.

In the pre-experiments, the number of poles and zeros was tried from 1 to 4 and 0 to 4, and the average value of *F P* reached a maximum when taking 3 and 1, respectively. Therefore, the transfer function in Eqs 3, 4 was specified in the following form:

$$H\left(s\right)=\frac{\alpha s+\delta }{s^{3}+As^{2}+Bs+C}e^{-Ts},$$ (9)

where *A*, *B*, *C*, *α*, and *δ* denote the free parameters to be estimated, and *T* represents the time delay. The time delay *T* in Eq. 9 was estimated by incrementally shifting in steps of 0.001 s from 0 to 5 s after the start of stimuli, until *A I C*, *B I C*, and *M S E* reached their minimum values as defined in Eqs 5, 7, 8 and *F P* reached its maximum value as defined in Eq. 6. This process aimed to identify the optimal transfer function with known dead time *τ* and peak time *T*_*p*_ as shown in Figure 3. The dead time of the impulse response, analogous to the onset latency in nerve conduction, represents the duration during which the index is unresponsive to the stimulus. The peak time, measured at the peak amplitude of the impulse response, corresponds to the peak latency Mallik and Weir (2005). The rise time *T*_*r*_, calculated by subtracting *τ* from *T*_*p*_, provides an indication of the time elapsed from peripheral activation to the maximum change in vascular tone or sweat rate, regardless of differences in nerve conduction velocity or neurotransmitter diffusion rate. Additionally, the waveform characteristics of the impulse response were also analyzed using the baseline-to-peak amplitude *A*_*p*_ in this study.

#### 2.4.2 Statistical analysis

All statistical tests presented in the manuscript are Brunner–Munzel test with Holm adjustment (significance level: 1%) unless otherwise noted. Cliff’s delta *δ* Cliff (1993) is presented as a measure of effect size ranging from −1 to +1. The absolute value of *δ* indicates the proximity of the two groups, while the sign of *δ* denotes whether group A is greater (+) or *vice versa* (−). The statistical package R, version 4.2.0 (R Foundation for Statistical Computing), was used in this study for statistical analysis.

## 3 Results

**FIGURE 4:**
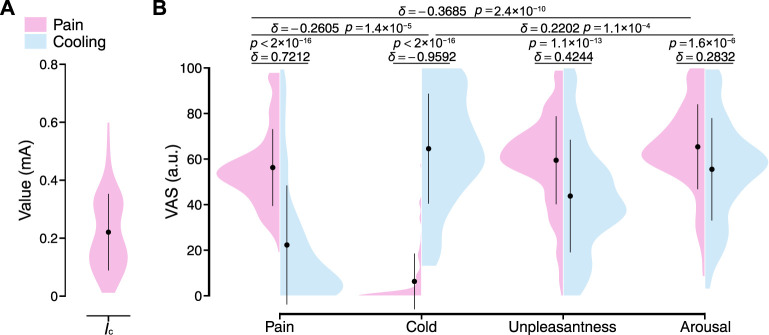
Stimulus intensities and resulting sensory changes for all participants. **(A)** Current intensity in the local pain stimulus experiment. *I*_*c*_: Current intensity. **(B)** The VAS ratings on pain, cold, unpleasantness, and arousal under local pain and local cooling stimuli. The black point range lines represent the mean and standard deviation. The statistical test results based on the effect size *δ* and the Brunner–Munzel test with Holm adjustment are also shown (significance level: 1%). VAS: Visual analog scale.

In this study, we collected time-series physiological data from the local pain and local cooling stimulus experiments to analyze the transient responses of the peripheral arterial stiffness index *β* across stimuli, aiming to assess its ability to characterize peripheral sympathetic activation. Figure 4A shows the stimulus intensities set in the local pain stimulus experiment for all participants, which were determined based on the participants’ verbal reports and ranged from 0.01 to 0.6 mA (0.22 ± 0.13 mA [mean ± S.D.]). As shown in Figure 4B, the local pain stimuli resulted in significant changes in pain sensation and unpleasantness, and no significant changes in cold sensation. The local cooling stimuli of 10°C provided sufficient cold sensation and also generated unpleasantness. However, some participants experienced painful sensations with the cold stimulus. Hence, the types (*δ* = 0.7212, *p* < 2 × 10^−16^; *δ* = −0.9592, *p* < 2 × 10^−16^) and valences (*δ* = 0.4244, *p* = 1.1 × 10^−13^) of sensations evoked by the two stimuli differed from each other, while only minor differences were found in the degree of sensory change (*δ* = −0.2605, *p* = 1.4 × 10^−5^). Moreover, both sensory stimuli were acute and strong enough to significantly arouse participants.

**FIGURE 5:**
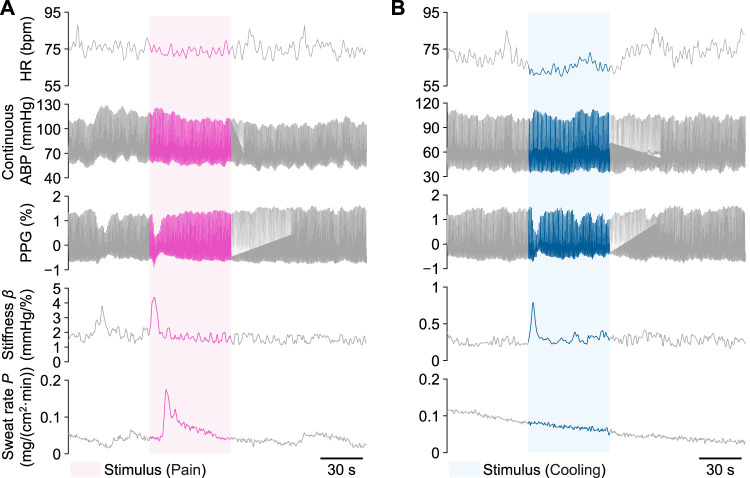
Examples of measured heart rate (HR), continuous arterial blood pressure (ABP), photoplethysmogram (PPG), peripheral arterial stiffness index *β*, and palm sweat rate *P* from Participant A during a single trial, respectively. **(A)** The local pain stimulus experiment. **(B)** The local cooling stimulus experiment. The colored areas indicate each 60-s stimulus period.

Figure 5 shows examples of time-series waveforms of the measured and calculated signals for Participant A in the local pain and local cooling stimulus experiments, respectively. In response to the local pain stimulus, a noticeable decrease in the amplitude of PPG was observed, accompanied by a significant increase in the amplitudes of the peripheral arterial stiffness index *β* and palm sweat rate *P*. In addition, there was a slight increase in the continuous ABP, while no significant change was observed in the amplitude of HR. Similarly, the local cooling stimulus led to a significant decrease in the amplitude of PPG and an elevation in the amplitude of *β*. There was also a slight increase in the continuous ABP and no significant change in the HR. However, unlike the changes in *P* during the local pain stimulus, the amplitude of *P* did not significantly change during the local cooling stimulus. Furthermore, distinct periodic fluctuations in HR and *β* amplitudes were observed, particularly during periods without stimuli.

**FIGURE 6:**
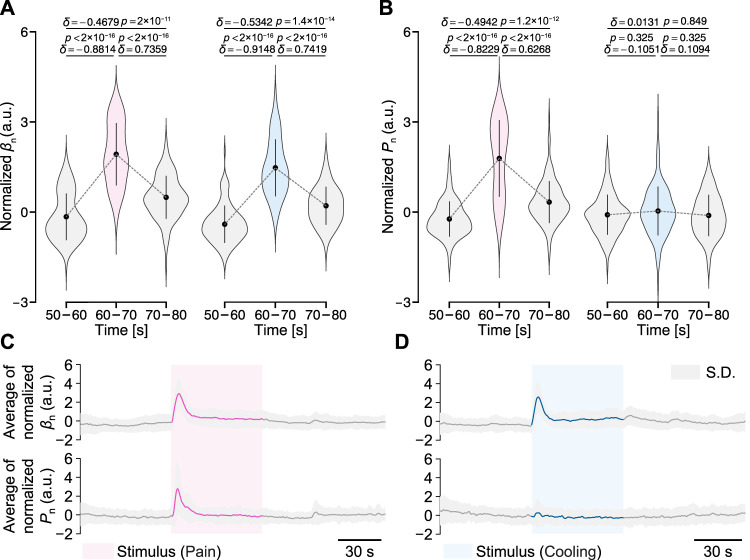
Normalized peripheral arterial stiffness *β*_n_ and palm sweat rate *P*_n_ for all participants in the two experiments. **(A,B)** Their mean values over different periods. The gray dashed lines represent their group-averaged changes. The black point-range lines represent the mean and standard deviation (S.D.). The statistical test results based on the effect size *δ* and the Brunner–Munzel test with Holm adjustment are also shown (significance level: 1%). **(C,D)** Group-averaged results of *β*_n_ and *P*_n_.

Figure 6 shows the normalized results of the peripheral arterial stiffness index *β* and palm sweat rate *P* for all participants. Figures 6A, B shows the changes in the mean values of normalized *β*_n_ and *P*_n_ at baseline (50–60 and 70–80 s) and during the period after the start of stimuli (60–70 s). For the peripheral arterial stiffness index, a significant increase was found between the pre-stimulus baseline and the period after the start of stimuli in both experiments (*δ* = −0.8814, *p* < 2 × 10^−16^; *δ* = −0.9148, *p* < 2 × 10^−16^). In both experiments, the value of *β*_n_ rapidly returned to the baseline after 10 s, which was moderately above the pre-stimulus baseline (*δ* = −0.4679, *p* < 2 × 10^−11^; *δ* = −0.5342, *p* < 1.4 × 10^−14^). For the palm sweat rate, a significant increase was found between the pre-stimulus baseline and the period after the start of stimuli only in the local pain stimulus experiment (*δ* = −0.8229, *p* < 2 × 10^−16^) and its value also returned to the baseline by the similar degree as above. However, in the local cooling stimulus experiment, the measured valuee did not change significantly over the three periods (*δ* = −0.1051, *p* = 0.325; *δ* = 0.1094, *p* = 0.325; *δ* = 0.0131, *p* = 0.849). Figures 6C, D show the group-averaged results of normalized *β*_n_ and *P*_n_ for all participants. Similarly, a significant increase can be found in the waveform of *β*_n_ in each experiment, while only in that of *P*_n_ in the local cooling stimulus experiment. In addition, differences in the baseline values of *β*_n_ and *P*_n_ each before the stimuli and after recovery could be observed.

**FIGURE 7:**
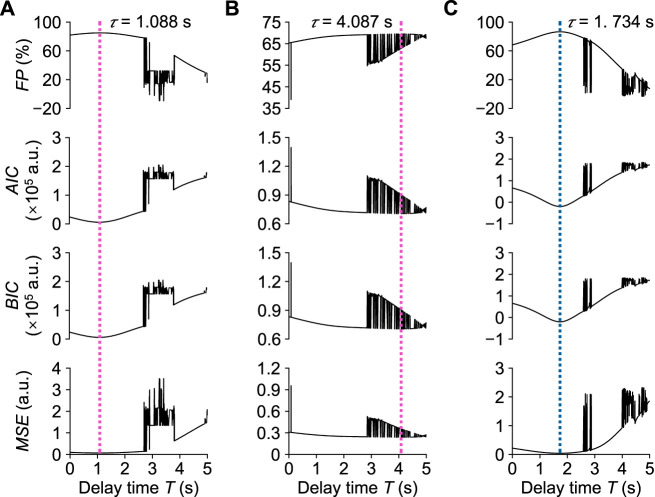
Assessment of transient response analysis results in one trial of Participant **(A)**. **(A)** Normalized *β*_n_ in the local pain stimulus experiment. **(B)** Normalized *P*_n_ in the local pain stimulus experiment. **(C)** Normalized *β*_n_ in the local cooling stimulus experiment. *τ*: Dead time.

Figure 7 shows the optimal results for the transient responses estimated by stepping the delay time *T* in one trial of Participant A. In the local pain stimulus experiment, the optimal transfer function $H_{\beta_{\text{p}}}\left(s\right)$ for *β*_n_ was identified as:

$$H_{\beta_{\text{p}}}\left(s\right)=\frac{8.97\;\times \;10^{-6}s+7.37\;\times \;10^{-11}}{s^{3}+8.18\;\times \;10^{-4}s^{2}+4.34\;\times \;10^{-7}s+6.35\;\times \;10^{-11}}e^{-1.088s}.$$ (10)

Here, when the time delay reached 1.088 s, i.e., the dead time *τ*, *F P* reached its maximum value of 85.02%, and *M S E*, *A I C*, and *B I C* reached their minimum values of 0.066, 5,832, and 5,903, respectively. The optimal transfer function $H_{P_{\text{p}}}\left(s\right)$ for *P*_n_ was identified as:

$$H_{P_{\text{p}}}\left(s\right)=\frac{-1.10\;\times \;10^{-5}s+4.83\;\times \;10^{-9}}{s^{3}+2.13\;\times \;10^{-3}s^{2}+7.87\;\times \;10^{-7}s+5.98\;\times \;10^{-10}}e^{-4.087s}.$$ (11)

Here, *F P* reached its maximum value of 69.49%, and *M S E*, *AIC*, and *BIC* respectively reached their minimum values of 0.240, 70,522, and 70,593 at the time delay of 4.087 s. In the local cooling stimulus experiment, the optimal transfer function $H_{\beta_{\text{c}}}\left(s\right)$ for *β*_n_ was identified as:

$$H_{\beta_{\text{c}}}\left(s\right)=\frac{8.89\;\times \;10^{-6}s-2.70\;\times \;10^{-11}}{s^{3}+1.26\;\times \;10^{-3}s^{2}+1.27\;\times \;10^{-6}s+3.40\;\times \;10^{-10}}e^{-1.732s}.$$ (12)

**FIGURE 8:**
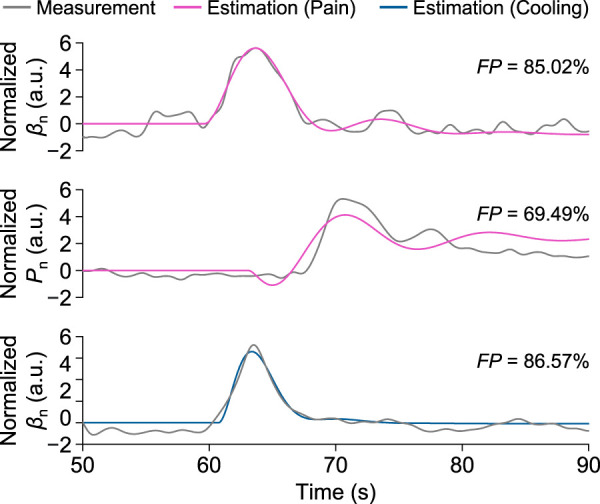
Measurement and estimation results of *β*_n_ and *P*_n_ for Participant A in one trial of the local pain and local cooling stimulus experiments, respectively.

Here, *F P* reached its maximum value of 86.57%, and *M S E*, *A I C*, and *B I C* respectively reached their minimum values of 0.039, −20,029, and −19,959 at the time delay of 1.732 s. Figure 8 shows the corresponding step responses based on the transfer functions identified above for Participant A in response to local pain and local cooling stimuli.

**FIGURE 9:**
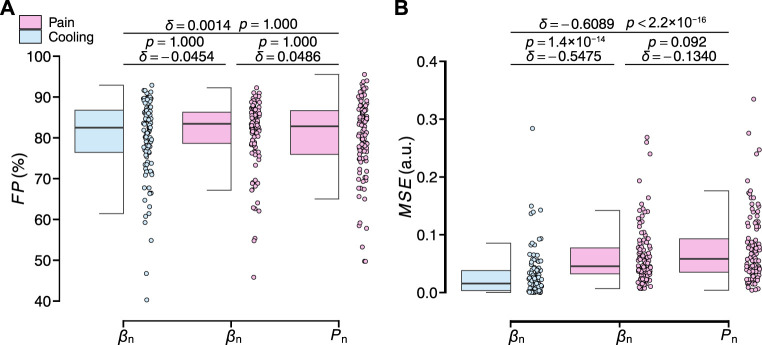
Evaluation results of the transient response analysis results of normalized *β*_n_ and *P*_n_ for all participants. **(A)** *F P*. **(B)** *MSE*. The statistical test results based on the effect size *δ* and the Brunner–Munzel test with Holm adjustment are also shown (significance level: 1%).

Figure 9 shows the evaluation results of the transient response analysis results among all participants. The transfer function can precisely characterize the transient responses of the three measured variables (*F P*: 79.92% ± 8.79%, 78.40% ± 11.00%, and 78.40% ± 10.90%). For the values of *M S E* between the three measured variables, those of *β*_n_ under the local cooling stimuli significantly differed from those of *β*_n_ and *P*_n_ under the local pain stimuli (*δ* = −0.5475, *p* = 1.4 × 10^−14^; *δ* = −0.6089, *p* < 2.2 × 10^−16^), while there was no significant difference between those of the latter two variables (*δ* = −0.1340, *p* = 0.092). All results of the identification of the free parameters of the model and the evaluation results of the transient analysis for all participants are summarized in Supplementary Table S1.

**FIGURE 10:**
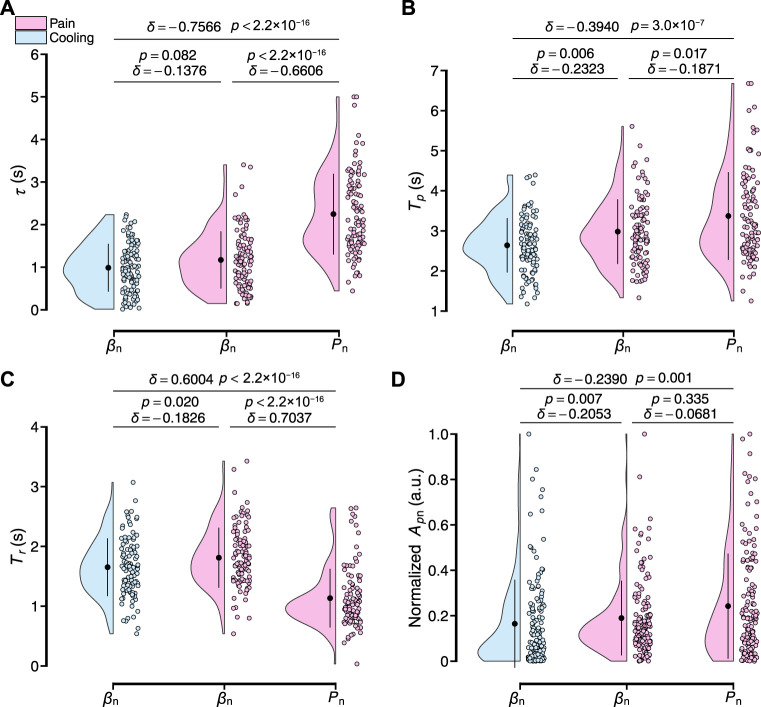
Results of the transient response characteristics of normalized *β*_n_ and *P*_n_ to local pain and local cooling stimuli for all participants. **(A–D)** Dead time *τ*, peak time *T*_*p*_, rise time *T*_*r*_, and baseline-to-peak amplitude *A*_*p*n_. The black point range lines represent the mean and standard deviation. The statistical test results based on the effect size *δ* and the Brunner–Munzel test with Holm adjustment are also shown (significance level: 1%).

Figure 10 shows the four characteristics parameters of the transient response analyzed in this study for all participants, including the dead time *τ*, peak time *T*_*p*_, rise time *T*_*r*_, and baseline-to-peak amplitude *A*_*p*_. For the dead time *τ*, the delay of the peripheral arterial stiffness index to the local pain and local cooling stimuli were respectively 1.17 ± 0.67 and 0.99 ± 0.56 s [mean ± S.D.] and differed from each other slightly (*δ* = −0.1376, *p* = 0.082). And that of the palm sweat rate to the local pain stimulus was 2.25 ± 0.95 s, significantly differing from those of the stiffness index. For the peak time *T*_*p*_, the values of the stiffness index to the local pain and local cooling stimuli were respectively 2.98 ± 0.81 s and 2.64 ± 0.68 s. And that of the palm sweat rate to the local pain stimulus was 3.37 ± 1.09 s, with a moderate difference from that of *β* for the local pain stimuli and significant difference from that of *β* for the local cooling stimuli. For the rise time *T*_*r*_, the values of the stiffness index to the local pain and local cooling stimuli were respectively 1.81 ± 0.50 s and 1.65 ± 0.48 s and both normally distributed (*p* = 0.757 and 0.116, Shapiro–Wilk normality test, significance level: 1%). And that of the palm sweat rate to the local pain stimulus was 1.13 ± 0.49 s, significantly shorter than those of the two variables of *β*. For the normalized baseline-to-peak amplitude *A*_*p*n_, a slight difference in the amplitudes was observed between *β* under the local cooling stimulus and the local pain stimuli (0.19 ± 0.16 and 0.16 ± 0.19 a.u), suggesting comparable vasomotor velocities when considering the difference in rise time. Therefore, it could be verified that the two sensory stimuli did elicit different patterns of peripheral sympathetic activation, as evidenced by the four transient characteristic parameters of *β*. In other words, the peripheral arterial stiffness index can serve as a noninvasive biomarker to characterize peripheral sympathetic activation.

## 4 Discussion

In this study, the ability of the peripheral arterial stiffness index to characterize peripheral sympathetic activation was investigated through experiments involving two local sensory stimuli. Here, transfer functions were separately constructed to model and describe the responses of the peripheral arterial stiffness index and palm sweat rate. The results of the transient analyses indicate that the index is effective in characterizing peripheral sympathetic activation, experimentally evidencing its potential to accurately assess stimulus cues and underlying pathologies.

### 4.1 Sensory basis for transient analysis

The two sensory stimuli used in our study elicited distinct sensations, potentially activating different cortical areas, which in turn could mediate differential sympathetic innervation of peripheral target organs Kandel et al. (2021); Craig and Bushnell (1994); Craig et al. (1996); Kop et al. (2011). Moreover, the notable differences in VAS ratings for the unpleasantness of each stimuli may also contribute to this differential sympathetic innervation Hamunen et al. (2012). The arousal that accompanies both sensory stimuli is a general response common to pain, cold, and other sensations, brought about by significantly elevated sensory changes that subsequently orient attention on stimuli and somatosensory detection Kuwahara et al. (2015); Rodenkirch et al. (2019); Lee et al. (2020). In particular, affective arousal can intensify autonomic arousal, particularly sympathetic activation, to modulate peripheral responses in target organs as parts of defensive mechanisms Lee et al. (2020); Allen et al. (2016); Satpute et al. (2019). Therefore, the two sensory stimuli designed in this study provided an experimental basis for investigating the characterization of the peripheral arterial stiffness index.

### 4.2 Neural basis for transient analysis

The immediate decrease in PPG amplitude and the corresponding increase in *β* amplitude following each stimulus confirmed the effectiveness of both sensory stimuli in inducing peripheral vasomotor responses. Nevertheless, direct evidence linking these two sensory stimuli to modulation of the peripheral vasculature through the SNS is lacking, as peripheral vasoconstriction can also be intensified by local chemicals and hormones Clifford (2011); Hall (2018). However, participants were kept in a supine position throughout the experiment, visual and auditory disturbances were isolated using shaded clothes and noise-canceling headphones, and the respiratory modulation of the SNS could be eliminated by a low-pass filter. Therefore, sympathetic activation was considered to arise only from the applied sensory stimuli or mental stress, as in the first resting period of the local pain stimulus experiment in Participant A. In the local pain stimulus experiment, the presence of palm sweating in a thermoneutral environment, without a significant increase in HR and ABP, suggests sympathetic activation due to sensory changes. This observation indirectly supports the hypothesis of parallel sympathetic control over sweating and vasoconstriction triggered by sensory stimuli. Further evidence arises from a previous study showing a positive correlation between changes in this index and pain-related brain activity Tsuji et al. (2021). Therefore, as shown in Figure 6, the significant response of the index to different sensory stimuli across all participants solidifies its ability to assess sympathetic activation.

For the palm sweat rate, the absence of significant changes in most participants following local cold exposure might be attributed to the suppression of sudorific neurotransmitter release and reduced sensitivity of receptors on sweat glands Okamoto et al. (1994); Grassi et al. (2003); Wingo et al. (2010). This lack of response could indicate a pattern of peripheral sympathetic activation distinct from that triggered by the local pain stimulus. Furthermore, the slight increase in ABP under both stimuli suggests extensive vasoconstriction in the peripheral vasculature, which prevents these two patterns of peripheral sympathetic activation from being classified as simply MSNA and SSNA Greaney and Kenney (2017); Bini et al. (1980); Mano (1998); Greaney et al. (2016); Ikäheimo (2018). Overall, both sensory stimuli in this study elicited peripheral sympathetic activation with two distinct patterns and modulated possibly different responses of *β*, the characterization ability of which can be accordingly verified by analyzing its transient characteristics.

### 4.3 Transient analysis results and relevant characteristics

The transfer function used in the transient analysis accurately characterized the responses of the peripheral arterial stiffness index and palm sweat rate, as evidenced by the fit of the estimated step response to the measures. However, since the dead time of *P*_n_ was considerably longer than that of two *β*_n_, and its *F P* was lower than that of *β*_n_ in Participant A, it may be necessary to extend the estimation interval beyond 5 s or investigate the applicability of the model to the palm sweat rate of all participants. Moreover, the inter- and intra-individual differences in transient responses of all participants were further confirmed from the differential estimates of free parameters and the corresponding evaluation results, thereby revealing distinctive transient response characteristics. Considering the high *F P* and low *M S E* values of the identification results for the three variables, the applicability of the pre-constructed model to describe the transient responses across all participants was demonstrated. Therefore, a transfer function with three poles, one zero, and a delay time can accurately describe the transient response of both *β* and *P* to the sensory stimuli in this study. Consequently, the identified impulse response could function in characterizing and assessing the differential patterns of peripheral sympathetic activation across participants.

The observed variation in peak amplitude of the peripheral arterial stiffness index *β* indicates comparable vasomotor velocities in response to different stimuli. This implies that the time taken for vascular smooth muscle cells to contract from a tonic level to their maximum is relatively consistent for stimuli with similar intensity and frequency Eccles and Wilson (1974). However, the high onset latency in the impulse response of *P* contrasts with previous findings, which may be attributed to the digital perspiration meter’s limited sensitivity in detecting sweat changes. An alternative approach, such as measuring the sympathetic skin response with an onset latency of 1.3–1.5 s at the hands, could offer more sensitivity Gutrecht (1994). Then, the sensitivity and time delay of the measurement hardware can also influence identification results, which can be ignored in this study for the same measurement site and method. In addition, local cold exposure is known to boost SSNA and may potentially elicit MSNA depending on stimulus intensity Kregel et al. (1992); Greaney et al. (2016); Sawasaki et al. (2001). Local pain stimuli applied to the skin typically elevate SSNA while causing a transient decrease in MSNA Burton et al. (2016). This pattern, combined with the observed unresponsiveness of the palm sweat rate to local cooling stimuli, leads to the hypothesis that MSNA might predominantly contribute to peripheral sympathetic activation elicited by local cooling stimuli in this study, while SSNA might predominate under local pain stimuli. Physiologically, the nerve conduction velocity of MSNA, recorded from the peroneal nerve, is about 1 m/s, while the vasoconstrictor and sudomotor bursts (SSNA), recorded from the tibial nerve, exhibit velocities of approximately 0.76 and 0.95 m/s, respectively Mano et al. (2006); Kondo et al. (2004). These differences in nerve conduction velocities may account for the variation in dead time. Overall, the different characteristics of the impulse responses to different stimuli underscore the index’s ability to characterize sympathetic activation.

### 4.4 Limitations of the study

In this study, a black-box model was constructed based on measured input-output pairs, wherein sensory changes were specified as the sole sympathetic activator without considering the physiological system’s role in regulating the vasomotor response. Meanwhile, the input should reflect the actual level of peripheral sympathetic activation, not an estimate derived from the peripheral arterial stiffness index. Despite designing two completely different sensory stimuli to evaluate the characterization ability of this index, the corresponding patterns of sympathetic activation, though distinguishable, could not be directly identified without simultaneous measurement of the microneurography. Furthermore, this study was unable to detect differences in nerve conduction properties, potentially due to the omission of factors like individual differences in height or limb length. Finally, as this index quantifies functional changes in arterial stiffness due to sympathetic activation, its applicability for pathological subjects with varying degrees of arterial stiffness (e.g., hypertensive patients) requires further investigation.

## 5 Conclusion

This study modeled the transient responses of the peripheral arterial stiffness index, revealing distinct timescale characteristics when subjected to the two different sensory stimuli. This distinction enabled the differentiation of various patterns of peripheral sympathetic activation. Notably, the observed differences in timescale and waveform characteristics also underscored the similarity in vasomotor velocity under both stimuli, further validating the index’s ability to characterize sympathetic activation. In summary, this study establishes a noninvasive characterization biomarker of peripheral sympathetic activation and provides a computational basis for developing a noninvasive and accurate evaluation technique for peripheral sympathetic activation.

While further sympathetic stimuli will be necessary to refine the validation of the peripheral arterial stiffness as a discriminative biomarker, it is also imperative to evaluate the applicability of the transfer function constructed in this study before characterizing other stimuli. Concurrently, integrating simultaneous measurements of the index with microneurography will be expected to provide direct evidence to identify patterns of peripheral sympathetic activation.

## Data Availability

The original contributions presented in the study are included in the article/Supplementary Material, further inquiries can be directed to the corresponding authors.
