# Supplementary material for: Noninvasive characterization of peripheral sympathetic activation across sensory stimuli using a peripheral arterial stiffness index
Source: Front Physiol. 2024 Jan 8;14:1294239. doi: 10.3389/fphys.2023.1294239 (PMC10801023; doi:10.3389/fphys.2023.1294239)
Supplement: Supplementary file 1 [file DataSheet1.PDF]

Supplementary Material

1 IDENTIFICATION RESULTS

This file contains Supplementary results of the identification of the free parameters of the model and the evaluation results of the transient analysis for all participants (Table S1). Among them, the mean values of the fit percent *FP* and mean squared error *MSE* of the transfer function in the analysis of the transient responses of the peripheral arterial stiffness to the two local sensory stimuli were 78.40±11.00% and 79.80±10.90%, and 0.068±0.092 and 0.070±0.055 [mean±S.D.], respectively. Those of the palm sweat rate to the local pain stimuli for all the participants were 79.92±8.79% and 0.029±0.040, respectively.

Table S1. Transient response analysis results of normalized β_{n} and normalized P_{n} to local pain and local cooling stimuli for all participants. S.D.: standard deviation.

		**Normalized β_{n} (Pain)**	**Normalized P_{n} (Pain)**	**Normalized β_{n} (Cooling)**
	**Items**	**mean [S.D.]**	**mean [S.D.]**	**mean [S.D.]**
Free parameters	α (a.u.)	8.35×10^{-5} [7.16×10^{-4}]	7.33×10^{-5} [1.81×10^{-4}]	1.96×10^{-6} [3.18×10^{-6}]
	δ (a.u.)	-2.17×10^{-9} [2.23×10^{-8}]	-2.68×10^{-9} [2.08×10^{-8}]	-2.51×10^{-11} [2.11×10^{-10}]
	δ/α (a.u.)	-1.79×10^{-5} [8.54×10^{-5}]	-7.96×10^{-6} [4.05×10^{-5}]	-1.38×10^{-5} [5.90×10^{-5}]
	A (a.u.)	3.41×10^{-2} [3.45×10^{-1}]	2.39×10^{-3} [6.18×10^{-3}]	8.94×10^{-4} [5.72×10^{-4}]
	B (a.u.)	1.34×10^{-5} [1.34×10^{-4}]	2.04×10^{-6} [4.46×10^{-6}]	6.21×10^{-7} [7.88×10^{-7}]
	C (a.u.)	2.50×10^{-9} [2.51×10^{-8}]	4.47×10^{-10} [1.13×10^{-9}]	1.23×10^{-10} [2.35×10^{-10}]
	T (s)	1.17 [0.67]	2.25 [0.95]	0.99 [0.56]
Evaluation indices	*FP* (%)	78.40 [11.00]	79.80 [10.90]	79.92 [8.79]
	MSE (a.u.)	0.068 [0.092]	0.070 [0.055]	0.029 [0.040]
	AIC (a.u.)	-1.24×10^{4} [4.36×10^{4}]	-6.89×10^{3} [4.33×10^{4}]	-8.60×10^{4} [8.92×10^{4}]
	BIC (a.u.)	-1.24×10^{4} [4.36×10^{4}]	-6.82×10^{3} [4.33×10^{4}]	-8.60×10^{4} [8.92×10^{4}]

2 IDENTIFIED IMPULSE RESPONSES

Figure S1 shows the group-averaged results of the normalized impulse responses β_{imp} and P_{imp} transformed from the estimated transfer functions of normalized β_{n} and P_{n} in response to the two local sensory stimuli for all participants. The first-order derivates $\dot{\beta}_{\mathrm{n}}$ and $\dot{P}_{\mathrm{n}}$ of corresponding group-averaged measurements of normalized β_{n} and P_{n} were also computed for comparison with the impulse responses. The time lags between β_{imp} and $\dot{\beta}_{\mathrm{n}}$, and P_{n} and $\dot{P}_{\mathrm{n}}$ in the local pain stimulus experiment, and between β_{imp} and $\dot{\beta}_{\mathrm{n}}$ in the local cooling stimulus experiments were -1.2, -1.1, and -0.9 s, respectively. Figure S1(B–D) shows strong linear correlations found between the normalized impulse responses with time lag adjustment and the first-order derivates with Pearson correlation coefficients of $r = 0.9790$, 0.9809, and 0.9575 ($p < 2.2 \times 10^{-16}$), respectively. The impulse responses of the three measurements manifested similar waveforms while featuring different characteristics. Moreover, by aligning the impulse responses on the time axis based on the calculated time lag, high degrees of similarity between each impulse response and the first-order derivative of its corresponding measurement can be observed, further confirming the accuracy of the identification results.

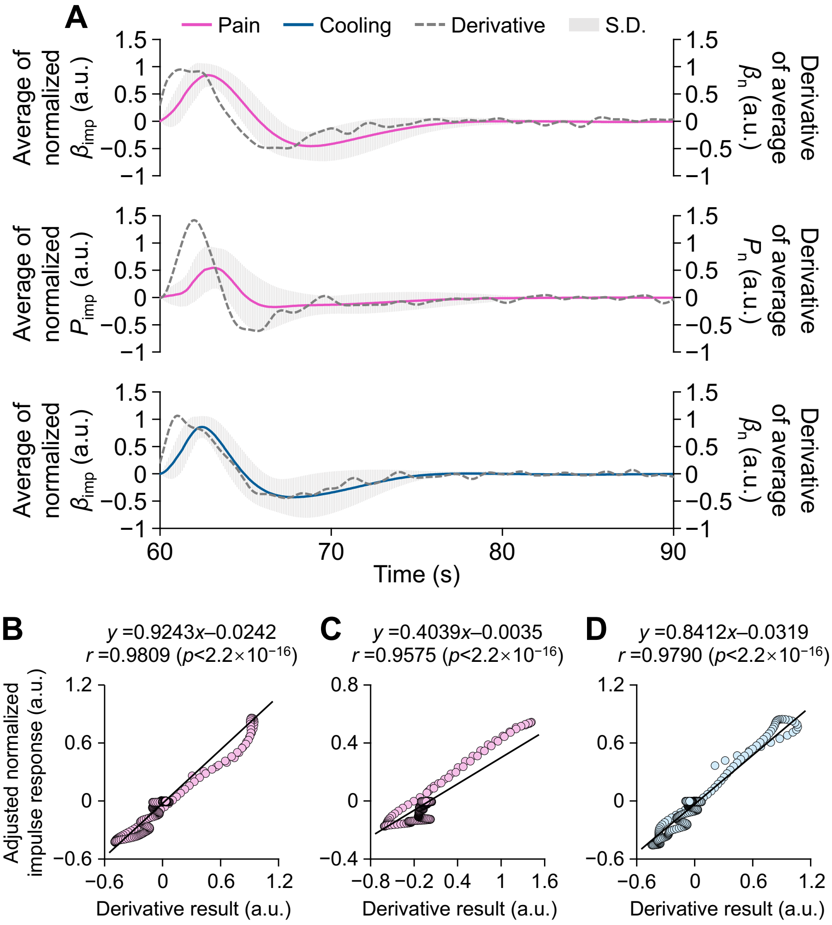

Figure S1. Group-averaged results of normalized impulse responses β_{imp} and P_{imp} to local pain and local cooling stimuli for all participants. **(A)** Waveform comparison. The gray dashed lines represent the first-order derivatives $\dot{\beta}_{\mathrm{n}}$ and $\dot{P}_{\mathrm{n}}$ of corresponding group-averaged measurements. S.D.: Standard deviation. **(B–D)** Results of Deming regression analysis on the first-order derivatives and normalized impulse responses with time lag adjustment. The statistical test results for Pearson correlation coefficient (significance level: 1%) are indicated by solid black lines.
